# Letter from Arizona

**Published:** 1882-05

**Authors:** W. G. Cook

**Affiliations:** Papago Indian Agency; Tucson, Arizona Territory


### Article XIV.

Papago Indian Agency, Feb. 24, 1882.

Messrs Editors:—I have just now returned from a visit to the Pima Indian Agency, and it may not be uninteresting to you to know something of them. They live on a Reserve on the Gila river, and are quite extensive farmers. Although they were primarily of the same nation as the Papagoes (my Indians) still they are different in a great many respects. There dress is quite summer-like in character, and they wear their hair long, and braided into small tails hanging down their backs. Their houses, or "kees," as they are called, are made of straw, with wooden frame work. There is a small door through which they crawl, and this furnishes the only entrance of air, or is the only means of ventilation. They have a curious door, made in the shape of a funnel, which admits the air, but does not allow its escape. I can hardly explain its philosophy. In these miserable hovels they live; eating, sleeping and sitting in the one room, for that is all they have.

The morals of these Indians, Pimas and Maricopas, are very loose. The Maricopas are fast dying out, being swept away by the ravages of venereal disease. The Pimas are not so bad, but still the virtue of their women is not above reproach. They have at the Agency a well-organized police force, composed of the best Indians in the tribe, who arrest all turbulent, drunken, or otherwise unruly Indians. These are tried by the Agent, and sentenced to different punishments according to his judgment. The police are furnished with arms and uniforms by the United States.

At the Agency the Government has established a boarding-school for the Indians, and its success is well established. They can accommodate 750 pupils. Food and clothing are furnished the children while at school. I forgot to mention that the police are furnished weekly rations and paid $5.00 per month besides their arms and clothing.

The Pimas have many songs, which are as rare in sentiment as they are in music and tune. I will endeavor to preserve some of them and send them to you, as they are curiosities. They have many superstitions, and hold many things sacred. They think the tails of different animals and birds are efficacious in driving away certain diseases. They have, however, no medicine-men.

I remain yours sincerely,

W. G. COOK.

*Tucson, Arizona Territory.*

